# Investigating the mediating effect of plasma metabolites on the gut microbiome in influencing Behçet disease: A multi-omics validated Mendelian randomization study

**DOI:** 10.1097/MD.0000000000042698

**Published:** 2025-08-08

**Authors:** Tao Guo, Lei Chang, Pan-Wang Huang, Jin-Ping Yao, Yi-Chen Zhang, Chun-Yan Ren, Chuan-Qing Bao

**Affiliations:** a Department of General Surgery, Affiliated Hospital of Jiangnan University, Wuxi, Jiangsu Province, China; b Laboratory of Genomic and Precision Medicine, Wuxi School of Medicine, Jiangnan University, Wuxi, Jiangsu Province, China.

**Keywords:** Behçet disease, mediation effect, Mendelian randomization, microbiota, plasma metabolites

## Abstract

Although an established correlation between gut microbiota (GM) and Behçet syndrome exists, the potential mediating involvement of plasma metabolites remains unclear. Using the most recent statistical data from genome-wide association studies conducted in 2024, we investigated the causal relationships between 473 GM taxa, 233 circulating metabolites, and Behçet syndrome (Behçet disease [BD]) through a 2-sample Mendelian randomization approach. This analysis was further supported by incorporating transcriptome and metagenomic data related to BD. A 2-step methodology was employed to evaluate the extent to which the effect of GM on BD is mediated through plasma metabolites. These results were subsequently validated in a separate validation set. Our Mendelian randomization results demonstrated correlations between various GM and the risk of Behçet syndrome. The potential link between GM and BD risk may be mediated through plasma circulating metabolite levels. Specifically, for every standard deviation, an increase in the abundance of *Turicibacter sp001543345* was correlated with a 403% increase in BD risk (odds ratio : 5.03 [95% confidence interval, 1.77–14.25]). Meanwhile, the cholesteryl esters to total lipids ratio in large very low-density lipoprotein and the total cholesterol to total lipids ratio in very large very low-density lipoprotein increased by 4%. The proportion of indirect effects is 3.026% and 3.338%, respectively. Our study established a causal link between distinct GM and BD and quantified the proportion of effects mediated through plasma metabolites. These findings provide further insights for the treatment of BD.

## 1. Introduction

Behçet disease (BD), also known as Behçet syndrome, is a chronic, systemic vasculitis characterized by oral and genital ulcers, ocular inflammation (such as uveitis), and skin lesions.^[1]^ Clinically, the etiology of BD is complex and not fully understood, making it difficult to target the root cause for treatment. Current therapies primarily focus on symptom relief and inflammation control using corticosteroids^[2]^ and immunosuppressants,^[3]^ which may have significant side effects.

The gut microbiota (GM) is pivotal for human health and well-being. It aids in food digestion, nutrient absorption,^[4]^ immune regulation,^[5]^ production of metabolites like short-chain fatty acids (SCFAs),^[6]^ maintenance of gut barrier integrity,^[7]^ and modulation of the nervous system through the “gut-brain axis.^[8]^” Dysbiosis of the GM is closely correlated with various diseases, including gastrointestinal disorders, metabolic diseases, immune-related diseases, and neurological conditions.^[9,10]^ Dysbiosis of the GM is closely correlated with various diseases, including gastrointestinal disorders, metabolic diseases, immune-related diseases, and neurological conditions.^[11–13]^

BD, a chronic inflammatory disorder, significantly alters the metabolism of glycerophospholipids (such as phosphatidylethanolamine) in the plasma of BD patients. These changes impact cell membrane structure and function, affecting cell signaling and immune responses.^[14]^ Furthermore, oxidative stress levels are markedly elevated,^[15]^ as evidenced by significantly increased levels of several polyunsaturated fatty acids ^[14,16]^ and malondialdehyde.^[17]^ This situation leads to cellular damage and exacerbates the inflammatory response.

A Mendelian randomization (MR) study is a powerful method in epidemiological research that utilizes genetic variations as instrumental variables (IVs) to determine causal relationships between exposure factors and diseases.^[18]^ MR studies are more effective in minimizing confounding factors and reverse causality compared to other observational research methods, as genetic variations are randomly assigned at conception and are independent of environmental influences.^[19]^ Therefore, MR studies offer more reliable causal inferences, essential for identifying cancer risk factors and potential therapeutic targets.^[20]^ Our objective is to utilize MR to establish the link between GM and BD while assessing the involvement of plasma metabolites in mediating this correlation.

## 2. Materials and methods

### 2.1. Study design

**Figure 1.**
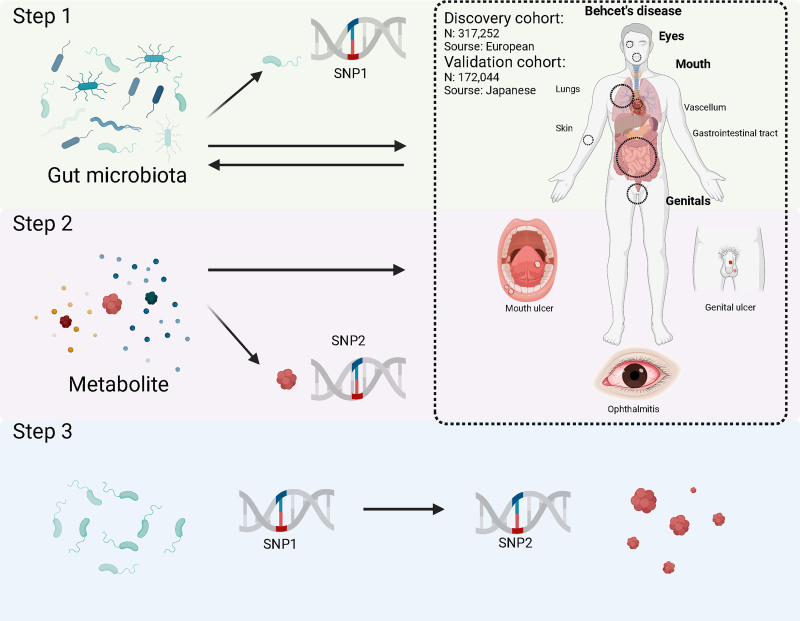
The study design of a 2-step MR analysis between GM and BD mediated by circulating metabolites. BD = Behçet disease, GM = gut microbiota, MR = Mendelian randomization, SNP = single nucleotide polymorphism.

This research utilized summary-level data from genome-wide association studies (GWAS) to conduct a 2-sample MR analysis, aiming to assess the relationships between GM, circulating metabolites, and BD. Our study strictly followed the 3 fundamental assumptions^[21]^ of MR: the IVs have a strong association with the exposure (relevance assumption); the IVs are not influenced by any confounders that could affect the relationship between exposure and outcome (independence assumption); and the IVs impact the outcome exclusively through the exposure, without any alternative pathways (exclusion restriction assumption).^[22]^ Research design is shown in Figure 1, which was created with *BioRender.com*.

### 2.2. Data source

Genetic variation data for GM were acquired from a large-scale cohort of 5959 genotyped individuals of European ancestry. After applying genome-wide significance thresholds, we identified 473 different taxa, including 11 phyla, 19 classes, 24 orders, 62 families, 146 genera, and 211 species.^[23]^ GWAS data for circulating metabolites were sourced from a study involving 33 cohorts comprising 1,36,016 participants primarily of European descent, encompassing 233 circulating metabolites.^[24]^ Discovery set data for BD are available from the GWAS catalog (GCST90018798), involving 3,17,512 samples and 1,90,84,009 single nucleotide polymorphism (SNP) loci. Validation set data for BD can also be obtained from the GWAS Catalog (GCST90018578), involving 1,72,044 samples and 1,24,53,729 SNP loci. Table 1 provides the details of these 4 datasets. This study used publicly published GWAS data sets for which all informed consent and ethical approval had previously been obtained. Therefore, no additional ethical approval for participation was required for this study.

**Table 1:** Behçet disease, intestinal microbiota and plasma metabolite data information and website.

Trait	ID	Year	Race	SNPs	Sample size	Web source
Gut microbiota	PMID: 35115689	2023	European	–	5959	https://www.ebi.ac.uk/gwas/publications/35115689
Plasma metabolites	PMID: 38448586	2024	European	–	1,36,016	https://www.ebi.ac.uk/gwas/publications/38448586
Behcet disease	ebi-a-GCST90018798	2021	European	1,90,84,009	3,17,252	https://www.ebi.ac.uk/gwas/publications/34594039
Behcet disease	ebi-a-GCST90018578	2021	East Asian	1,24,53,729	1,72,044	https://www.ebi.ac.uk/gwas/publications/34594039

SNPs = single nucleotide polymorphisms.

### 2.3. Instrumental variable

We selected genetic instruments based on specific criteria. Initially, SNPs showing significant association with the exposure factor (*P*-value <5 × 10^−8^) were retained as IVs. Given the limited SNPs in BD studies,^[25]^ we applied a *P*-value threshold of 5 × 10^−6^. Second, linkage disequilibrium was controlled by excluding SNPs with *r*^2^ < 0.001 within a physical distance of 10,000 kb to ensure the independence of IVs.^[26]^ Third, harmonization methods were utilized to remove palindromic and incompatible SNPs, and the *F*-statistic was applied to mitigate weak instrument bias. The *F* value was calculated using the formula: F=R2(N-K-1)K (1-R2), where *R*^2^ represents the cumulative variance explained by the exposure, *K* is the total number of IVs, and N is the total number of samples included in the GWAS. Each SNP’s *R*^2^ was computed as *R*^2^ = β^2^ × 2 × MAF × (1 − MAF), where β is the effect size estimate for each allele, and MAF represents the minor allele frequency.^[27]^ SNPs with an *F*-statistic <10 were excluded to ensure the robustness of the genetic instruments.^[28]^ Ambiguous palindromic SNPs were then removed through harmonization. Detailed information on the IVs was supplied in Tables S1–S3, Supplemental Digital Content, https://links.lww.com/MD/P600.

### 2.4. Transcriptome analysis

To assess the role of GM and plasma metabolites in the development of BD, we performed transcriptome analysis. RNA sequencing data comes from GEO database (Access numbers: GSE205867, GSE17114, GSE209567; https://www.ncbi.nlm.nih.gov/geo). Then, we conducted an analysis using the OECloud web server (https://cloud.oebiotech.cn/).

### 2.5. Microbial metagenomic analysis

To verify the changes in the abundance of *Turicibacter* and other bacteria in BD patients, we performed a microbial metagenomic analysis. The data of microorganisms were obtained from the National Center for Biotechnology Information database (Access number: PRJNA431482; https://www.ncbi.nlm.nih.gov/pmc/articles/PMC6091101/#MOESM1), and we performed linear discriminant analysis effect sizes to identify differentially abundant taxa in the BD group. Linear discriminant analysis was used to determine the discriminative features. An abundance difference tests were performed using prime software.

## 3. Mendelian randomization analysis

### 3.1. Preliminary analysis

We conducted bidirectional 2-sample MR to investigate the causal relationship between GM and BD. To ensure robustness and assess MR effects, we employed multiple methodologies. The primary method used was the inverse-variance weighted (IVW) approach, which assumes all SNPs are valid IVs and provides precise estimates. Additionally, we utilized MR-Egger, weighted median (WM) methods, and Bayesian weighted MR, which account for different assumptions regarding instrument validity. The WM method, in particular, can produce robust predictions even if up to 50% of the data comes from invalid instruments.^[29]^ MR-Egger uses the slope coefficient from Egger regression, offering valuable causal insights while detecting potential biases from small study effects.^[30]^ In Bayesian weighted MR, a unified statistical framework has been developed to address the uncertainties associated with estimated weak effects in GWAS, as well as the influence of horizontal pleiotropy.^[31]^ For MR of circulating metabolites, we use the same IV criteria and analytical methods.

### 3.2. Intermediary Mendelian randomization analysis

We also employed a 2-step MR approach to perform mediation analysis, examining if plasma metabolites mediate the pathway from GM to BD. The overall effect was broken down into indirect and direct effects. Specifically, the influence of GM on BD can be divided into 2 parts: the direct effect of GM on BD and the indirect effect mediated through plasma metabolites. We evaluated the mediated effect by calculating the ratio of the indirect effect to the total effect and then determined the 95% confidence intervals (CI) for these estimates.^[19]^

### 3.3. Analysis of pleiotropy and heterogeneity

To ensure the robustness of the statistically significant causal relationships, we performed several sensitivity analyses. We used Cochran’s *Q* statistic to assess heterogeneity, and the MR-Egger intercept analysis to evaluate pleiotropy.^[32]^ Additionally, a systematic leave-one-out analysis was carried out to examine the impact of individual SNPs on the study outcomes. The MR Pleiotropy RESidual Sum and Outlier (MR-PRESSO) method was employed to identify and exclude influential outliers.^[33]^ Consistency in *P*-values across the IVW and all 3 MR methods was required to confirm causal relationships. A *P*-value of <.05 was observed, indicating significant heterogeneity and pleiotropy in the sensitivity analyses. Cochran’s *Q* statistic was applied to examine the heterogeneity among the IVs.^[34]^ MR-PRESSO analysis was employed to exclude SNPs contributing to heterogeneity. The leave-one-out analysis identified potentially influential SNPs. The intercept from MR-Egger regression was used to assess pleiotropy. All analyses were performed utilizing R software (version 4.3.0) with the “TwoSampleMR” R package (version 0.5.7).^[35]^ The results were reported following the STROBE-MR guidelines (Strengthening the Reporting of Observational Studies in Epidemiology utilizing Mendelian Randomization).^[36]^ The results of the sensitivity analysis can be found in Table S4, Supplemental Digital Content, https://links.lww.com/MD/P600.

## 4. Result

### 4.1. Transcriptome changes in BD

**Figure 2.**
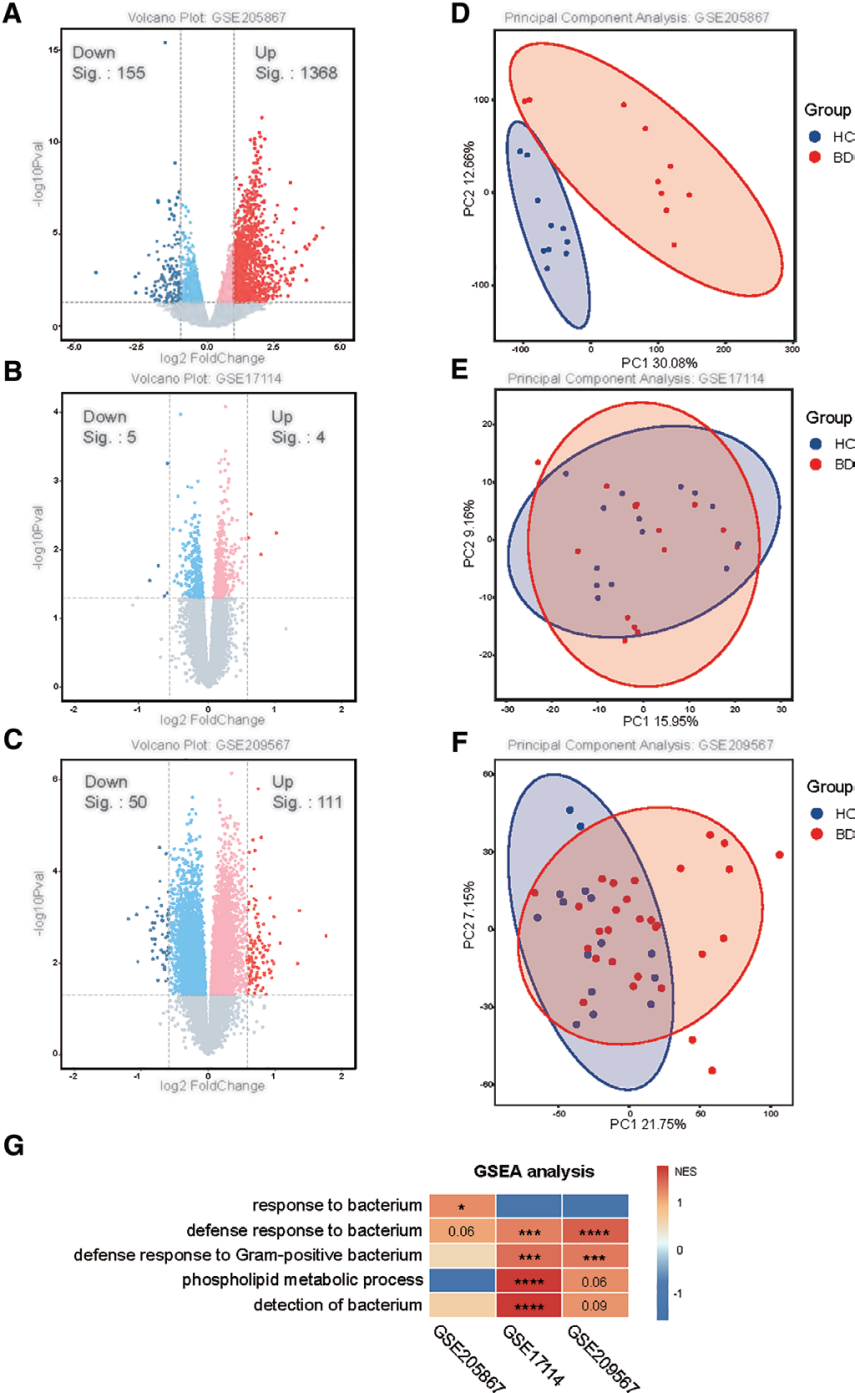
Three transcriptomic analysis results. (A–C) Volcanic map displays differentially expressed genes (DEGs). (D–F) PCA results of BD group and control group. (G) Heatmap of GSEA evaluation of response to bacterial and lipid metabolism pathways. BD = Behçet disease, DEGs = differentially expressed genes, GSEA = gene set enrichment analysis, HC = healthy control, PC = principal components, PCA = principal component analysis.

To investigate the relationship between BD and GM and plasma metabolites, we analyzed the gene expression changes in 3 groups of BD and healthy control patients. The volcano map shows significant changes in the genes of BD patients (Fig. 2A–C). The principal component analysis results also showed significant changes in the transcriptome of BD (Fig. 2D–F). In addition, we observed upregulation of pathways related to bacterial response and lipid metabolism in BD (Fig. 2G), indicating that GM and lipid metabolism may play a key role in the pathogenesis of BD.

### 4.2. The correlation between GM and BD

**Figure 3.**
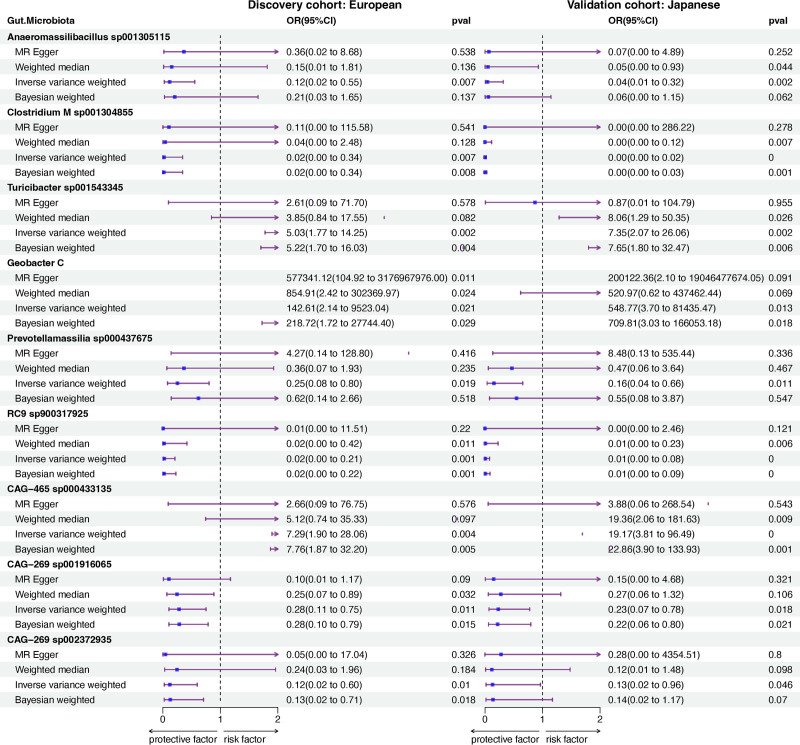
Mendelian randomization results of causal effects between gut microbiota and BD. BD = Behçet disease, CAG = *Clostridium* sp. CAG, CI = confidence interval, MR = Mendelian randomization, OR = odds ratio, RC = Rikenellaceae-rc9.

In the discovery cohort, we identified 23 gut microbes associated with BD, while 16 gut microbes were found to be associated with BD in the validation cohort. Nine gut microbes were common between the 2 cohorts (Fig. S1A, Supplemental Digital Content, https://links.lww.com/MD/P601). Among the 9 identified GM associated with BD risk, IVW and WM methods analyses revealed specific microbial abundances linked to decreased or increased BD risk (Fig. 3). Microbiota such as *CAG-269 sp001916065* (European [EUR]: IVW: OR = 0.28, 95% CI: 0.11–0.75, *P* = .011; JAP: IVW: OR = 0.23, 95% CI: 0.07–0.78, *P* = .018) and *Clostridium M sp001304855* (EUR: IVW: OR = 0.02, 95% CI: 0.00–0.34, *P* = .007; JAP: IVW: OR = 0.00, 95% CI: 0.00–0.02, *P* = .001) were associated with reduced BD risk, whereas *Geobacter C* (EUR: IVW: OR = 142.61, 95% CI: 2.14–9523.04, *P* = .021; JAP: IVW: OR = 548.77, 95% CI: 3.70–81435.47, *P* = .013) and *Turicibacter sp001543345* (EUR: IVW: OR = 5.03, 95% CI: 1.77–14.25, *P* = .002; JAP: IVW: OR = 7.35, 95% CI: 2.07–826.06, *P* = .002) were linked to increased BD risk. Reverse MR analysis did not find evidence of causal relationships between these microbiota and BD, as detailed in Table S5, Supplemental Digital Content, https://links.lww.com/MD/P600.

### 4.3. The correlation between plasma metabolites and BD

**Figure 4.**
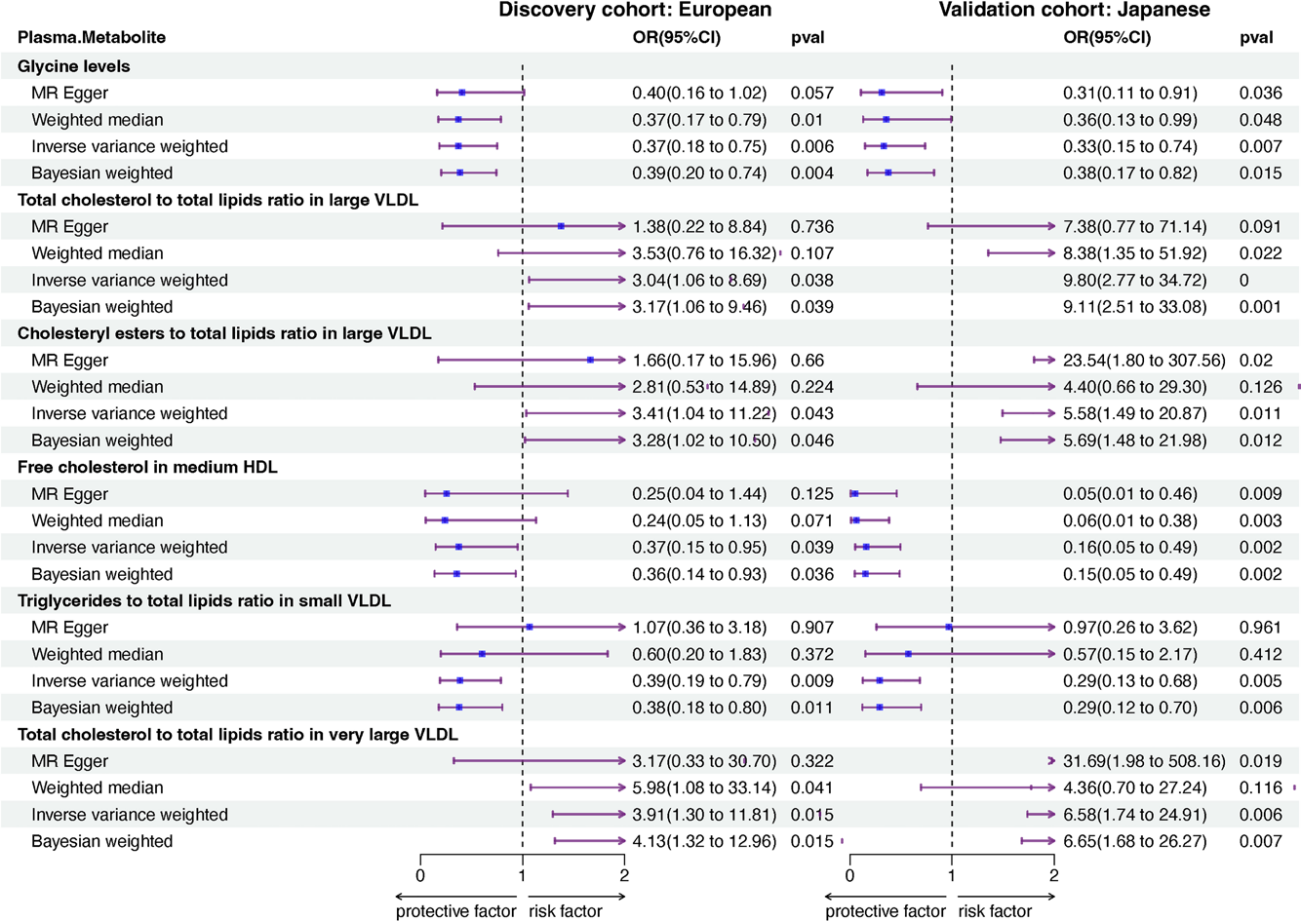
Mendelian randomization results of causal effects between circulating metabolites and BD. BD = Behçet disease, CI = confidence interval, HDL = high-density lipoprotein, MR = Mendelian randomization, OR = odds ratio, VLDL = very low-density lipoprotein.

In the MR analysis of circulating metabolites and BD, 6 metabolites were identified in the discovery cohort, and 19 metabolites were associated with BD in the validation cohort. Of these, 6 metabolites were included for further analysis (Fig. S1B, Supplemental Digital Content, https://links.lww.com/MD/P601). Notably, 3 metabolites showed positive associations with BD risk: the total cholesterol to total lipids ratio in large very low-density lipoprotein (VLDL; EUR: IVW, OR = 3.04, 95% CI: 1.06–8.69, *P* = .038; JAP: IVW, OR = 9.80, 95% CI: 2.77–34.72, *P* = .001), cholesteryl esters to total lipids ratio in large VLDL (EUR: IVW, OR = 3.41, 95% CI: 1.04–11.22, *P* = .043; JAP: IVW, OR = 5.58, 95% CI: 1.49–20.87, *P* = .011), and total cholesterol to total lipids ratio in very large VLDL (EUR: IVW, OR = 3.91, 95% CI: 1.30–11.81, *P* = .015; JAP: IVW, OR = 6.58, 95% CI: 1.74–24.91, *P* = .006). Conversely, 3 metabolites were negatively correlated with BD risk: glycine levels (EUR: IVW, OR = 0.37, 95% CI: 0.18–0.75, *P* = .006; JAP: IVW, OR = 0.33, 95% CI: 0.15–0.74, *P* = .007), free cholesterol in medium high-density lipoprotein (EUR: IVW, OR = 0.37, 95% CI: 0.15–0.95, *P* = .039; JAP: IVW, OR = 0.16, 95% CI: 0.05–0.49, *P* = .002), and triglycerides to total lipids ratio in small VLDL (EUR: IVW, OR = 0.39, 95% CI: 0.19–0.79, *P* = .009; JAP: IVW, OR = 0.29, 95% CI: 0.13–0.68, *P* = .005). However, glycine levels exhibited heterogeneity (*P* = .027), and triglycerides to total lipids ratio in small VLDL (*P* = .018) showed pleiotropy. Therefore, these 2 metabolites were excluded from further investigation (Fig. 4).

### 4.4. The correlation between GM and plasma metabolites

**Figure 5.**
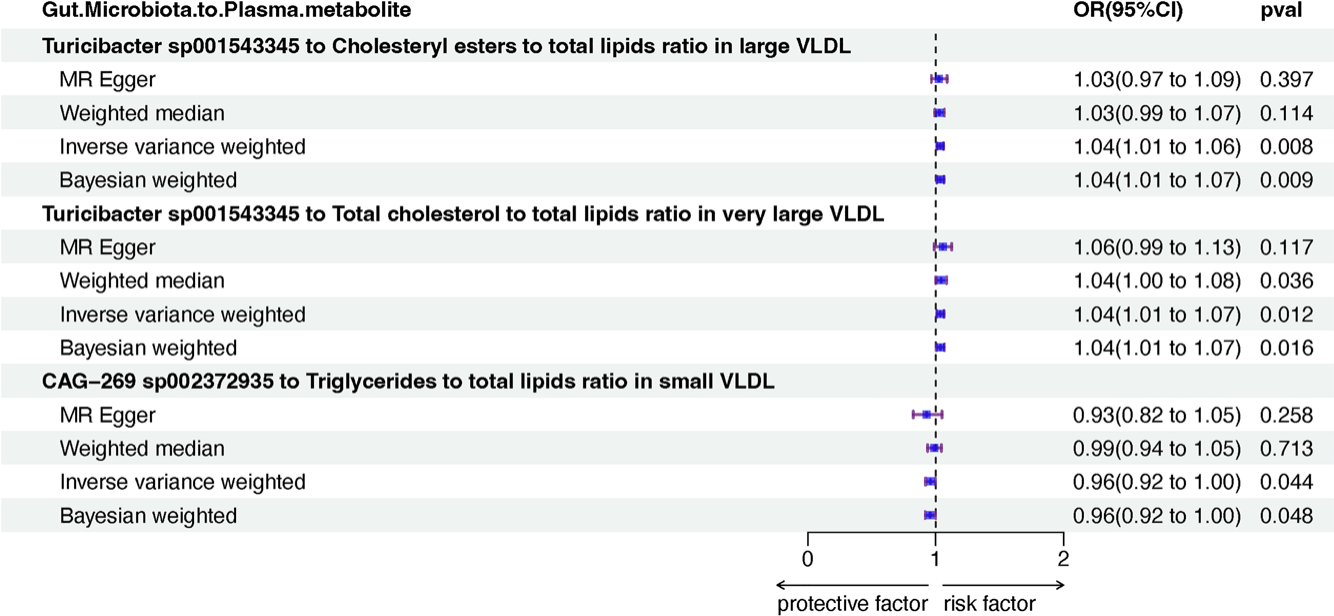
Mendelian randomization results of causal effects between gut microbiota and circulating metabolites. CI = confidence interval, CAG = *Clostridium* sp. CAG, MR = Mendelian randomization, OR = odds ratio, VLDL = very low-density lipoprotein.

In our MR analysis, we used SNPs associated with 9 GM species related to BD risk and SNPs related to 6 circulating metabolites. We identified 2 associations involving 1 GM species and these 2 circulating metabolites (Fig. S1C, Supplemental Digital Content, https://links.lww.com/MD/P601). An increase in *Turicibacter* sp001543345 abundance in stool was correlated with elevated levels of cholesteryl esters to total lipids ratio in large VLDL (IVW, OR = 1.04, 95% CI: 1.01–1.06, *P* = .008) and total cholesterol to total lipids ratio in VLDL (IVW, OR = 1.04, 95% CI: 1.01–1.06, *P* = .014; Fig. 5).

### 4.5. Mediation analysis results

**Figure 6.**
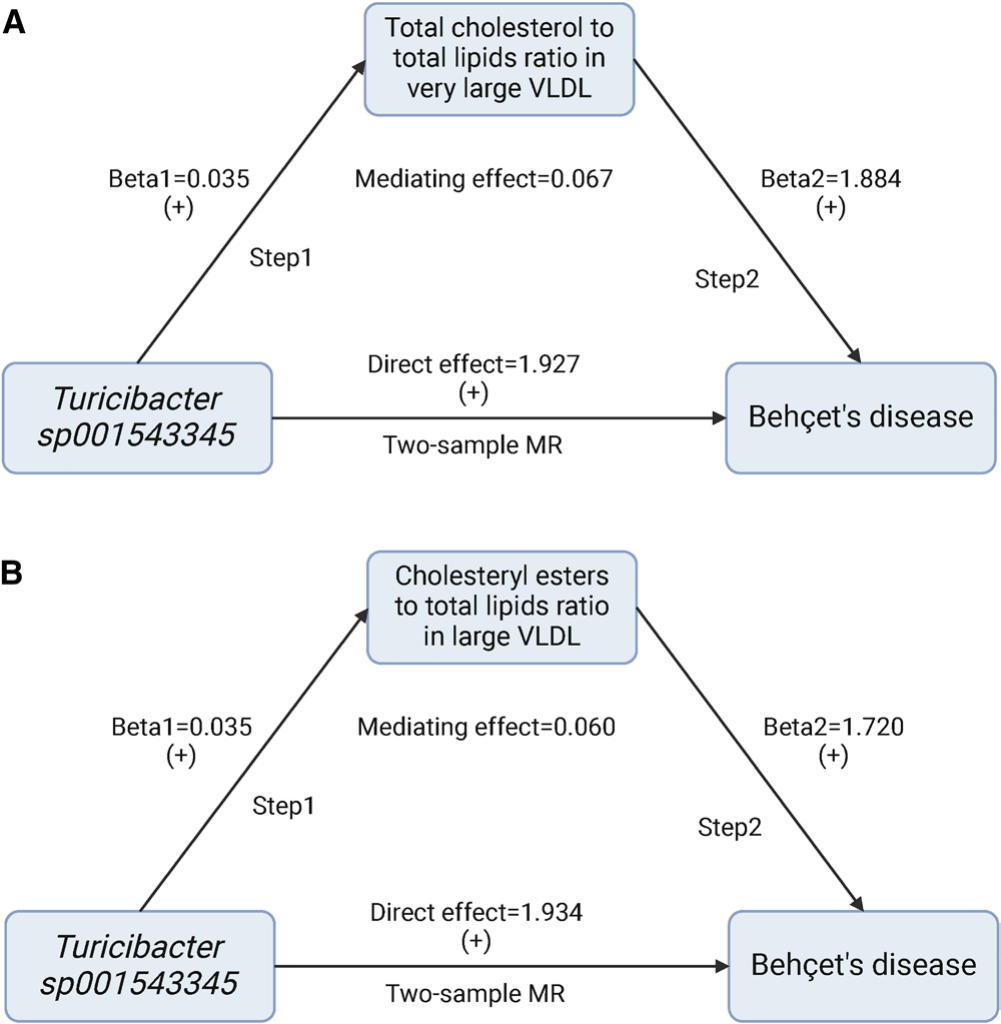
The result of mediation analysis. (A) The mediating effect of total cholesterol to total lipids ratio in very large VLDL between *Turicibacter* sp001543345 and Behçet disease is 6.7%. (B) The mediating effect of cholesteryl esters to total lipids ratio in large VLDL between *Turicibacter* sp001543345 and Behçet disease is 6%. MR = Mendelian randomization, VLDL = very low-density lipoprotein.

Based on the OR values between intestinal microbiota and circulating metabolites, we focused on *Turicibacter sp001543345* abundance and its relationship with total cholesterol to total lipids ratio in large VLDL and cholesteryl esters to total lipids ratio in large VLDL for mediation analysis. The effect size, derived from the beta coefficient using the IVW method, was examined. Through a 2-step MR analysis, we computed the potential mediating effect of the cholesteryl esters to total lipids ratio in large VLDL (mediation proportion = 3.026%, 95% CI = −110.69% to 116.74%) and total cholesterol to total lipids ratio in very large VLDL (mediation proportion = 3.338%, 95% CI = −122.47% to 129.14%) in the causal relationship between *Turicibacter sp001543345* abundance and BD risk (Fig. 6).

Our findings indicated that each standard deviation increase in *Turicibacter sp001543345* abundance corresponds to a 403% increase in BD risk (OR: 5.03, 95% CI: 1.77–14.25), while the cholesteryl esters to total lipids ratio in large VLDL increases by 4% (OR: 1.04, 95% CI: 1.01–1.06). Additionally, each standard deviation increase in the cholesteryl esters to total lipids ratio in large VLDL corresponds to a 241% elevation in BD risk (OR: 3.41, 95% CI: 1.04–11.22). We also observed that for every one standard deviation increase in *Turicibacter sp001543345* abundance, the total cholesterol to total lipids ratio in very large VLDL increased by 4% (OR: 1.04, 95% CI: 1.01–1.07). Additionally, each standard deviation increase in the total cholesterol to total lipids ratio in very large VLDL was associated with a 291% increase in BD risk (OR: 3.91, 95% CI: 1.30–11.81).

Our findings suggest that the cholesteryl esters to total lipids ratio in large VLDL and the total cholesterol to total lipids ratio in very large VLDL act as positive feedback regulators in the relationship between GM and Behçet syndrome. Increased abundance of *Turicibacter sp001543345* leads to elevated plasma levels of the cholesteryl esters to total lipids ratio in large VLDL and the total cholesterol to total lipids ratio in very large VLDL, thereby increasing BD risk factors.

### 4.6. Changes in gut microbiota in BD

**Figure 7.**
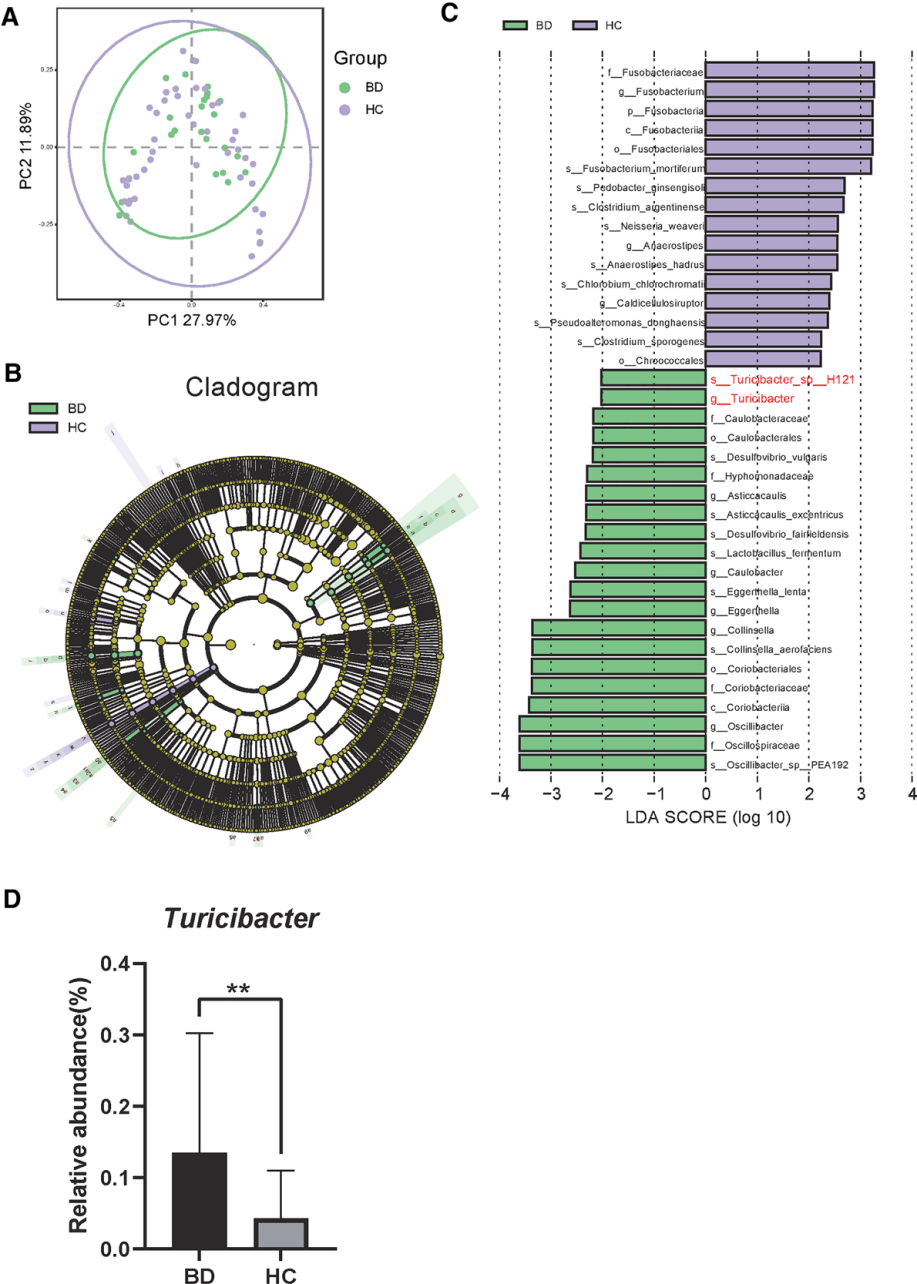
The results of metagenomic analysis (A) PCA result of BD and control group. (B) Differential species score plot illustrates taxonomic groups with LDA scores >2, which are significant between the 2 groups. (C) The LEfSe method identifies differential biomarkers at various taxonomic levels between the 2 groups. (D) The abundance changes of *Turicibacter* in BD group. BD = Behçet disease, HC = healthy control, LDA = linear discriminant analysis, LEfSe = linear discriminant analysis effect sizes, PC = principal components, PCA = principal component analysis.

To validate the aforementioned findings, we analyzed metagenomic sequencing data of GM from BD patients in the National Center for Biotechnology Information database. Principal component analysis results indicated significant alterations in the GM composition of the BD group compared to healthy controls (Fig. 7A). Additionally, linear discriminant analysis effect sizes analysis revealed that *Turicibacter* was significantly enriched in the BD group (Fig. 7B, C), with a relative abundance notably higher than in the control group (Fig. 7D). This suggests that *Turicibacter* may play a crucial role as a core microbe in the development of BD.

### 4.7. Sensitivity analysis and visualization results

We employed MR methods to conduct MR-Egger tests on the included SNP loci, with intercepts close to 0 (*P *> .05), and utilized MR-PRESSO tests to remove outlier SNPs (*P* > .05). The funnel plot illustrates the results of the heterogeneity assessment. Adjusted Cochran’s *Q* statistics indicated that the impact of the included SNPs did not exhibit significant heterogeneity (*P *> .05). Additionally, sensitivity analysis was performed utilizing the leave-one-out method to examine the impact of each SNP locus on the overall causal relationship. Upon sequentially removing each SNP and conducting reanalysis, no significant changes were noted in the established causal relationships. Heterogeneity tests and assessment of horizontal pleiotropy are reported in the supplementary materials, Supplemental Digital Content, https://links.lww.com/MD/P600. Scatter plots illustrated the trends in effects derived from various parameter estimation methods in GM (Fig. S2, Supplemental Digital Content, https://links.lww.com/MD/P601) and metabolites (Fig. S5, Supplemental Digital Content, https://links.lww.com/MD/P601). Funnel plot shows the results of heterogeneity assessment in GM (Fig. S3, Supplemental Digital Content, https://links.lww.com/MD/P601) and metabolites (Fig. S5, Supplemental Digital Content, https://links.lww.com/MD/P601) with IVW and MR-Egger methods. The leave-one-out plot illustrates the intensity of association between each SNP and the outcomes examined in GM (Fig. S4, Supplemental Digital Content, https://links.lww.com/MD/P601) and metabolites (Fig. S6, Supplemental Digital Content, https://links.lww.com/MD/P601).

## 5. Discussion

In this study, we utilized the latest GWAS databases to establish a potential causal correlation between 9 GM taxa and BD. Furthermore, bidirectional 2-sample MR analyses indicated that the association between GM and BD risk may be mediated by levels of 6 circulating metabolites. Our findings suggest that the abundance of *Turicibacter sp001543345* is linked to increased BD risk (mediation proportion = 3.026% and 3.338%). Additionally, this taxon’s detrimental effect is regulated through positive feedback on the total cholesterol to total lipids ratio in very large VLDL and the cholesteryl esters to total lipids ratio in large VLDL. This study represents the first exploration of plasma metabolites mediating interactions between GM and Behçet syndrome.

Our study revealed that an increased abundance of *Turicibacter sp001543345* is correlated with increased BD risk. Li et al conducted MR analyses on GM and BD without incorporating metabolites. They identified the genus *Parasutterella*, *Lachnospiraceae NC2004* group, *Turicibacter*, and *Erysipelatoclostridium* as protective factors for BD, while *Intestinibacter* was identified as a risk factor.^[37]^ Unfortunately, they did not specify the species within these genera. In contrast, our study utilized the latest published GWAS databases to identify 473 distinct taxonomic GMs causally associated with BD. Furthermore, we controlled for exposure and outcome data from European populations, effectively mitigating winner’s curse bias due to sample overlap,^[38]^ and further validated our findings using data from East Asian populations.

Behçet syndrome is a complex inflammatory disease affecting multiple systems, characterized by recurrent oral ulcers, genital ulcers, skin lesions, and ocular inflammation.^[39]^ Its pathogenesis is closely linked to genetic susceptibility, immune system dysregulation, and environmental factors.^[40]^ Its pathogenesis is closely linked to genetic susceptibility, immune system dysregulation, and environmental factors,^[41,42]^ ERAP1, IL10, and IL23R, which regulate immune responses.^[43,44]^ Furthermore, elevated levels of pro-inflammatory cytokines TNF-α, IL-6, IL-8, and IL-17 lead to immune system hyperactivation,^[45–47]^ increased proportions of Th1 and Th17 cells, and continuous secretion of pro-inflammatory cytokines.^[48]^ These processes sustain inflammation and contribute to neutrophil dysfunction, characterized by chemotaxis impairment and reactive oxygen species generation. Collectively, these factors promote tissue damage and exacerbate inflammation in Behçet syndrome.^[49]^

The intestinal microbiota regulates host immune responses through various mechanisms, including enhancing physical barriers,^[50]^ modulating innate^[51,52]^ and adaptive immunity,^[53]^ generating metabolites,^[54]^ and recognizing microbial-associated molecular patterns.^[55]^ These interactions are crucial for maintaining immune balance in the gut. Intestinal epithelial cells form physical barriers through tight junctions to prevent pathogen invasion. Microbial surface molecules, such as lipopolysaccharides and peptidoglycans, are recognized by pattern recognition receptors in the gut, activating immune cells like macrophages and dendritic cells to produce pro-inflammatory cytokines.^[56]^ Simultaneously, intestinal microbiota ferments dietary fibers to produce SCFAs like acetate, propionate, and butyrate. These metabolites activate G protein-coupled receptors (e.g., GPR41, GPR43), modulating immune cell functions. SCFAs also promote the generation of regulatory T cells (Tregs), suppress pro-inflammatory cytokine production, and exhibit anti-inflammatory properties.^[57]^ SCFAs also promote the generation of regulatory T cells (Tregs), suppress pro-inflammatory cytokine production, and exhibit anti-inflammatory properties.^[58–60]^ Additionally, the intestinal microbiota transforms primary bile acids into secondary bile acids, which regulate gut inflammation and systemic immune responses by activating the farnesoid X receptor and the G protein-coupled receptor TGR5.^[61]^

Multiple studies indicate significantly reduced GM diversity in patients with Behçet syndrome compared to healthy individuals. This reduction may impair the functional capacity of the GM to maintain gut health and balance.^[11,56]^ A study of Behçet syndrome patients in Japan found significantly reduced GM diversity, particularly in key probiotic populations such as *Megamonas hypermegale* and *Butyrivibrio*, essential for maintaining gut barrier function and suppressing inflammatory responses. Conversely, an increased abundance of potentially pathogenic bacteria such as *Lactobacillus* and *Bifidobacterium* is associated with gut inflammation and abnormal immune responses.^[62]^ Gut dysbiosis may compromise gut barrier function, increase intestinal permeability, and allow incompletely digested food components, toxins, and microbes to cross the intestinal barrier into the bloodstream, activating systemic immune responses. A study observed significantly weakened gut barrier function and increased intestinal permeability in Behçet syndrome patients,^[11]^ likely due to decreased beneficial and increased harmful bacteria.

Studies have shown that *Turicibacter* is associated with the development of colorectal cancer, with potential mechanisms involving the promotion of chronic inflammation, DNA damage, and the production of bioactive carcinogenic metabolites, which are elevated in colitis animal models and colorectal cancer patients.^[63,64]^ Different Turicibacter strains differentially affect host metabolites, including lipids and bile acids.^[65]^ Future research could further explore the use of Turicibacter and/or its bile modifications to purposefully alter host lipid biology, offering promising avenues for improving host metabolism and lipid-related health.^[66,67]^

Behçet syndrome patients commonly exhibit increased oxidative stress and lipid metabolism disorders, with elevated levels of total cholesterol, low-density lipoprotein cholesterol, and triglycerides in plasma, while high-density lipoprotein cholesterol levels are reduced.^[68]^ Oxidative stress drives elevated lipid peroxidation in BD patients, which disrupts cell membrane integrity and generates highly bioactive molecules, further promoting inflammatory responses and tissue damage. Chronic inflammation, a major feature of Behçet syndrome, can disrupt lipid metabolism pathways through various mechanisms.^[69,70]^ Inflammatory cytokines can regulate lipid metabolism pathways, leading to abnormal lipid levels. Conversely, lipid metabolism abnormalities can exacerbate inflammation by affecting cell membrane lipid composition and generating inflammatory mediators.^[49,71]^ Thus, we hypothesize that identifying effective drugs or targets to diminish the total cholesterol to total lipids ratio in large VLDL levels could potentially decelerate the progression of Behçet syndrome.

This study represents the first application of MR to explore causal links among GM, plasma metabolites, and BD. Our approach incorporated various sensitivity analyses to effectively mitigate the impact of confounding factors and reverse causality. Initial findings indicate a causal link between GM and BD involving intermediate factors. These results offer theoretical support for novel approaches in BD treatment and prevention. For instance, the management of BD can be tailored to individual patient presentations and rely on glucocorticoids, colchicine, and conventional or biologic immunosuppressants, as well as methods to modulate specific GM. Additionally, coordinated regulation of plasma metabolite levels may offer promising avenues for advancing BD prevention and treatment.

MR serves as a robust causal inference technique for pinpointing genetic variants linked to the research objective, effectively mitigating confounding influences and reverse causality.^[72]^ Nonetheless, our theoretical findings necessitate validation via clinical or animal studies to elucidate the underlying mechanisms. Further research involving cellular and animal models is essential for this purpose. Ultimately, we aim to validate our findings through population-level randomized clinical trials.

## 6. Conclusion

Our mediation analysis using MR found a causal relationship between GM, plasma metabolites, and Behçet syndrome. Specifically, *Turicibacter sp001543345* regulates BD through the metabolic pathway involving the total cholesterol to total lipids ratio in very large VLDL and the cholesteryl esters to total lipids ratio in large VLDL in plasma. Our study provides genetic evidence highlighting the interconnections among GM, plasma metabolites, and BD. These findings suggest that future interventions could focus on enhancing GM and pharmacologically modulating the lipid changes in plasma to enhance prevention and treatment strategies for BD patients.

## Acknowledgments

We’d like to extend our gratitude to the research community for making GWAS summary statistics publicly accessible. We extend our heartfelt gratitude to all the clinical patients who participated in this study and generously provided their valuable sample data, which significantly contributed to the successful completion of our research.

## Author contributions

**Data curation:** Tao Guo, Pan-Wang Huang, Jin-Ping Yao.

**Formal analysis:** Pan-Wang Huang, Jin-Ping Yao.

**Funding acquisition:** Chuan-Qing Bao.

**Investigation:** Lei Chang.

**Validation:** Lei Chang.

**Writing – original draft:** Tao Guo.

**Writing – review & editing:** Yi-Chen Zhang, Chun-Yan Ren, Chuan-Qing Bao.

## Supplementary Material


