# Supplementary material for: Investigating the mediating effect of plasma metabolites on the gut microbiome in influencing Behçet disease: A multi-omics validated Mendelian randomization study
Source: Medicine (Baltimore). 2025 Aug 8;104(32):e42698. doi: 10.1097/MD.0000000000042698 (PMC12338280; doi:10.1097/MD.0000000000042698)

Fig S1. Venn diagram shows overlapping bacteria and metabolites between the discovery cohort and validation cohort.

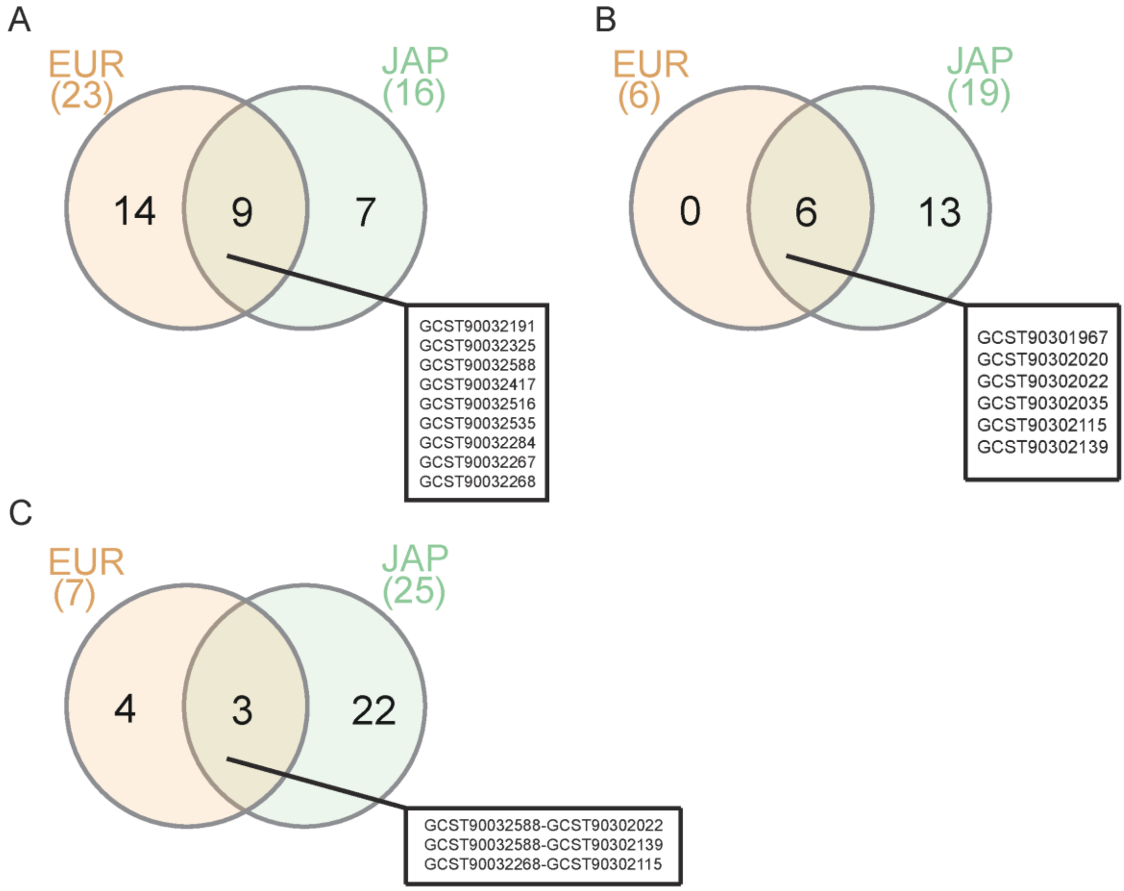

Fig S2. Scatter plot between Gut Microbiota to BD.

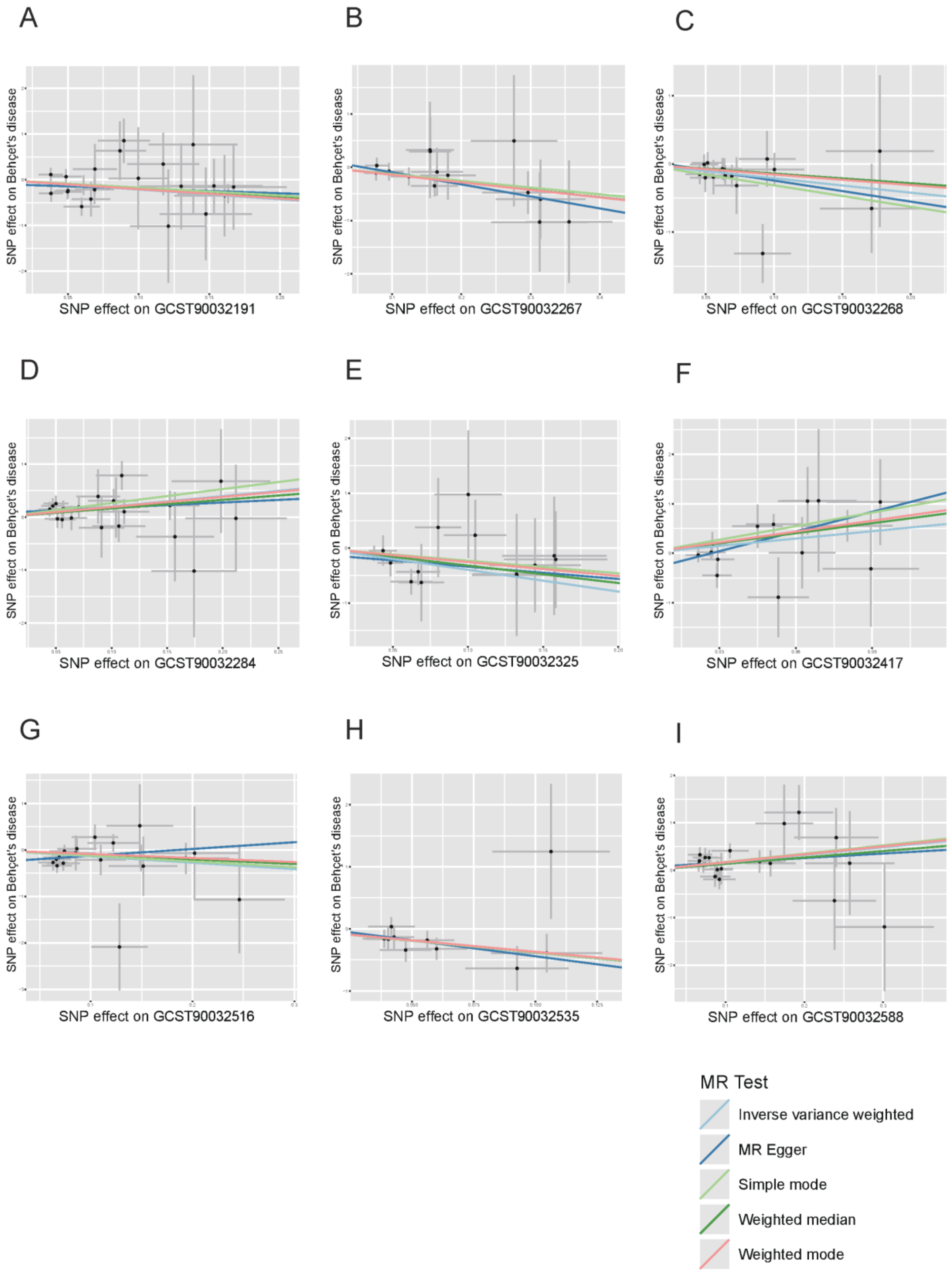

Fig S3. Funnel plot testing for heterogeneity between Gut Microbiota to BD.

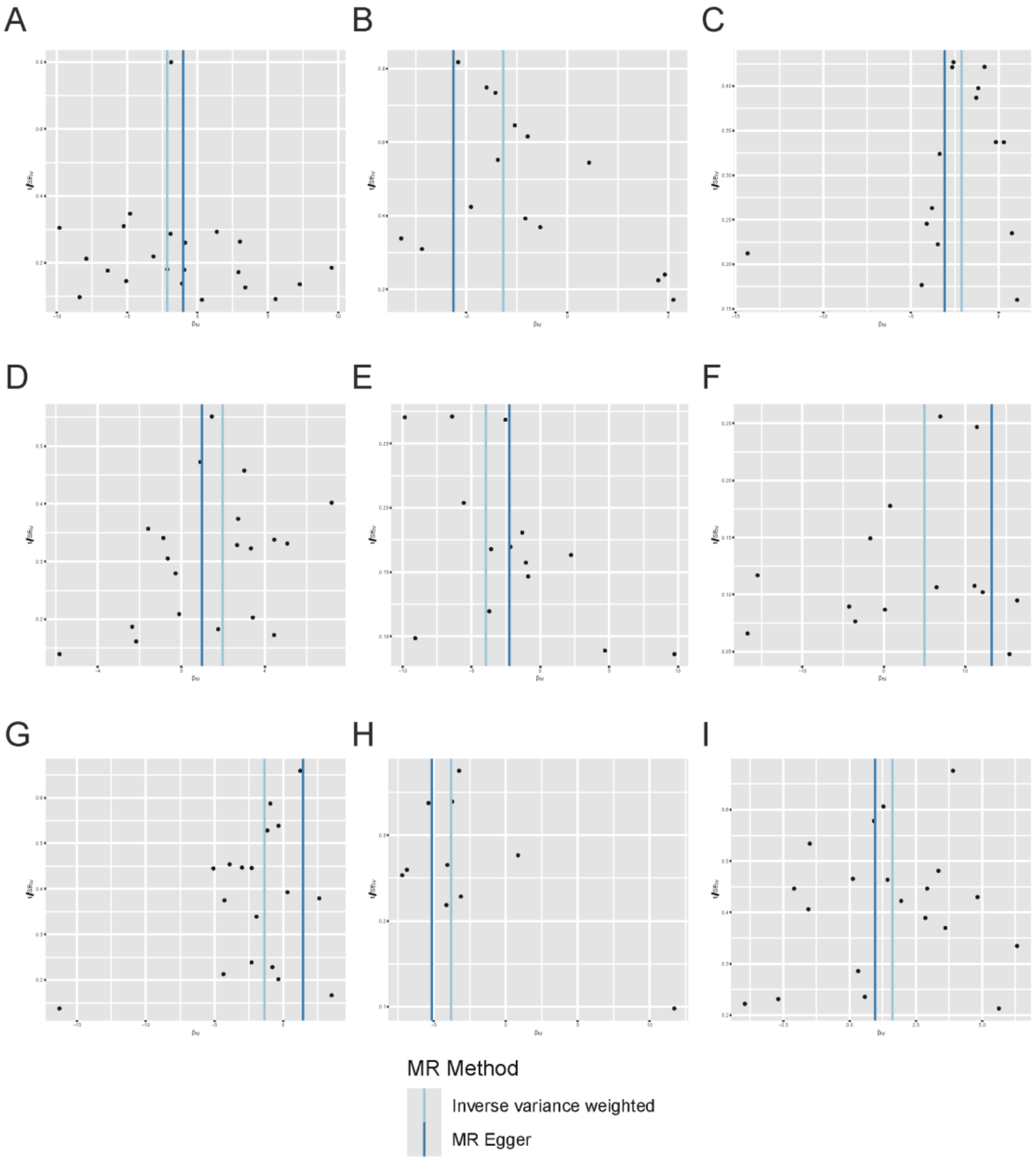

Fig S4. Leave-one-out results illustrating the intensity of each SNP in the analysis between Gut Microbiota and BD.

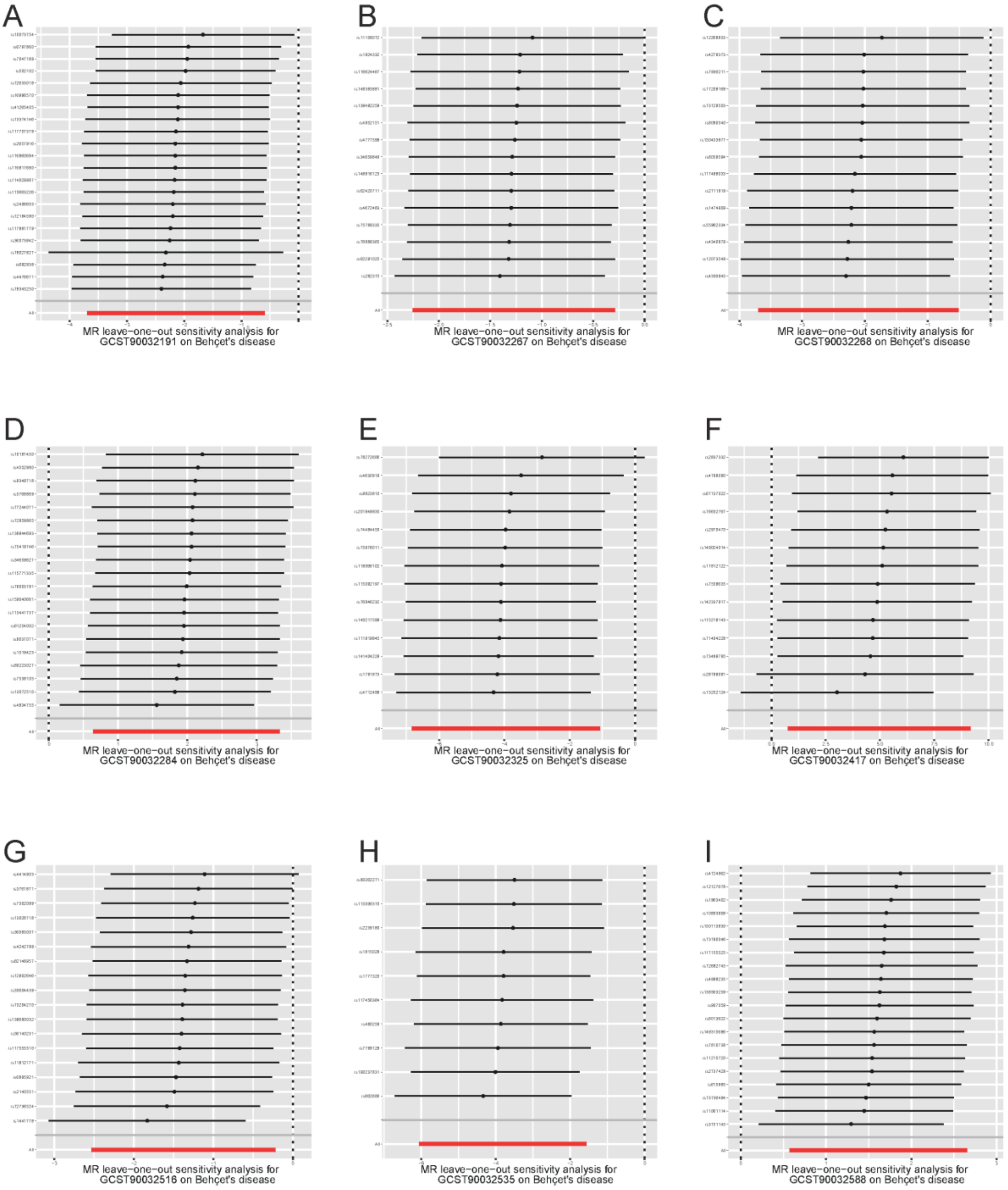

Fig S5. Scatter plot and Funnel plot between circulating metabolites to BD.

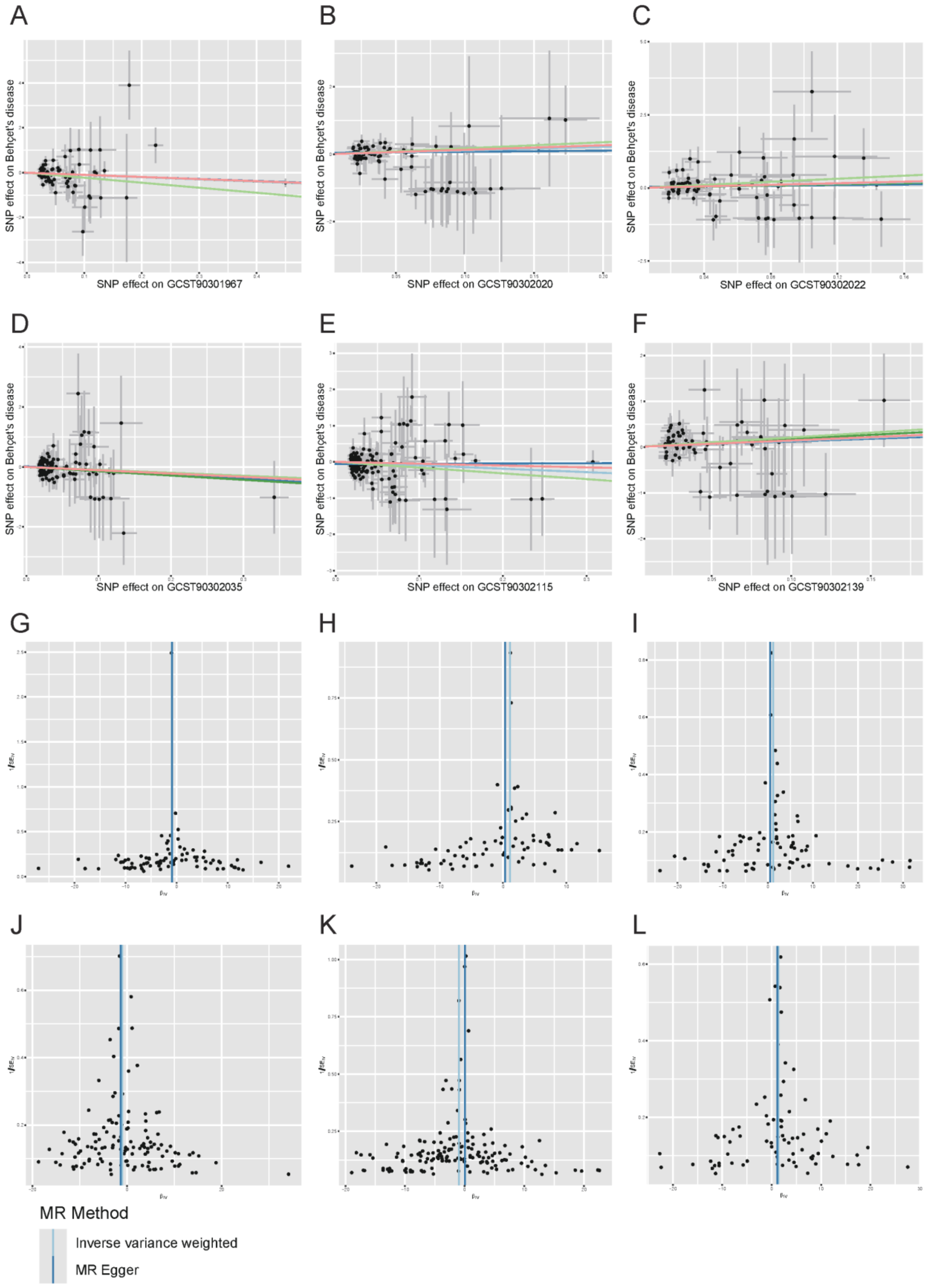

Fig S6. Leave-one-out results between circulating metabolites to BD.

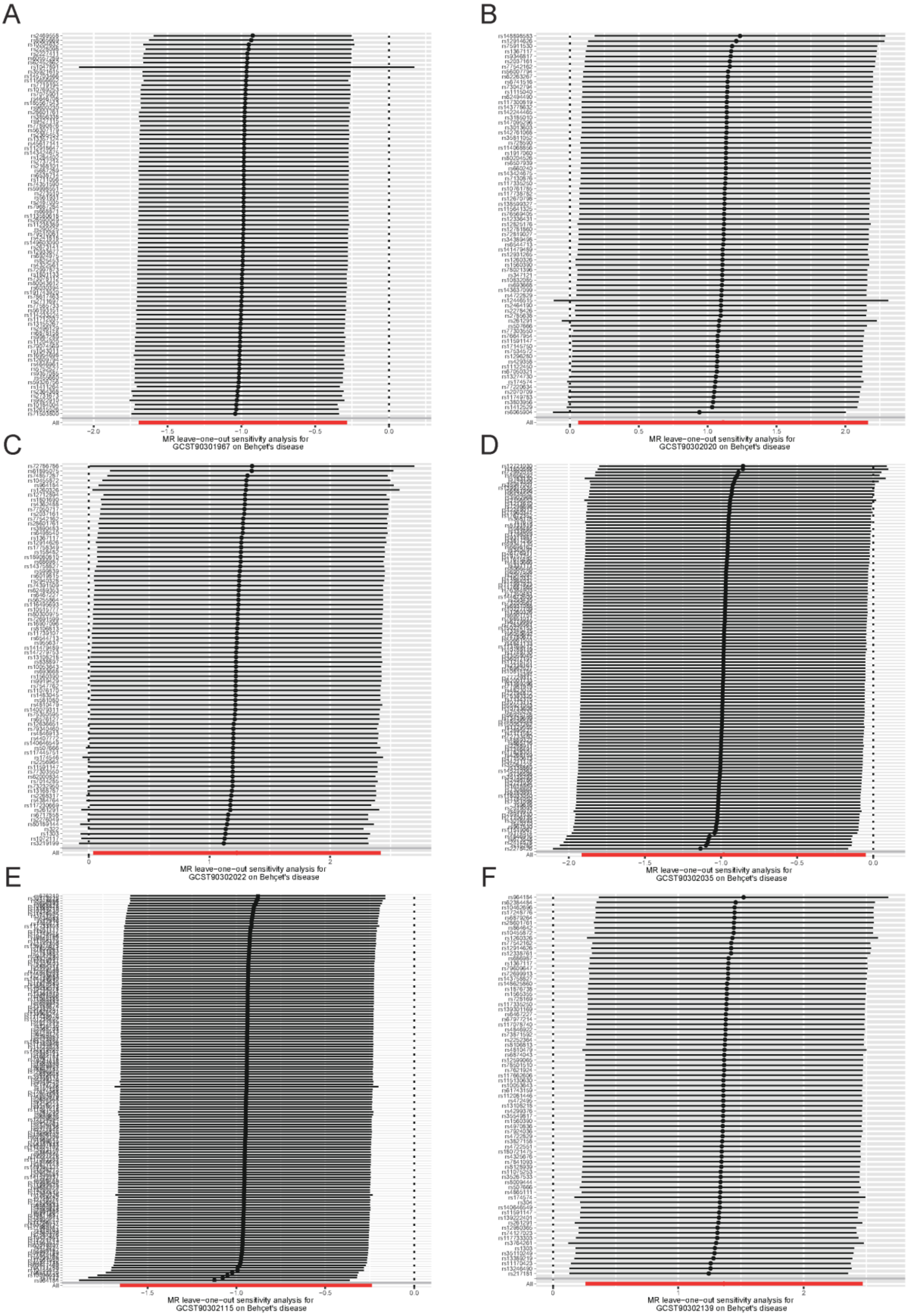

Supplement: Supplementary file 2 [file medi-104-e42698-s002.pdf]
